# Outcome of Allogeneic Adult Stem Cell Therapy in Dogs Suffering from Osteoarthritis and Other Joint Defects

**DOI:** 10.1155/2018/7309201

**Published:** 2018-06-28

**Authors:** Kiran Shah, Tara Drury, Ivelise Roic, Peter Hansen, Mark Malin, Richard Boyd, Huseyin Sumer, Ray Ferguson

**Affiliations:** ^1^Australian Veterinary Stem Cell P/L, Melbourne, Australia; ^2^Swinburne University of Technology, Hawthorn, Australia; ^3^Hudson Institute, Clayton, Australia; ^4^Monash Vet Clinic, Clayton, Australia

## Abstract

Osteoarthritis is a common condition that causes joint pain and stiffness that affects both humans and dogs. In Australia, allogeneic canine adipose-derived mesenchymal stem cells for therapy have been commercially available since 2010. In this report, we describe the outcome of the treatment of two hundred and three dogs diagnosed with degenerative arthritis with severe chronic pain in joints causing lameness at walk, reduced mobility, and functional disability. Posttreatment assessment data after 10 weeks revealed significant improvement (*p* < 0.007) of the symptoms: pain reduction, improvement of mobility, and increased daily activity as measured as quality of life score. Ninety percent of young dogs (<9 years) showed excellent improvement in pain and mobility and were able to run and resume normal activity. Sixty percent of older dogs showed good improvement. However, 12% of dogs did not exhibit any change in symptoms; one dog showed worsening of the symptoms. This report provides the support for the safety and efficacies of allogeneic adipose-derived mesenchymal stem cells in a regenerative therapeutic veterinary model.

## 1. Introduction

Osteoarthritis (OA) is a common degenerative disease in dogs and is a major cause of disability. It is estimated that one in five dogs in Australia and the US suffers from this debilitating disease, which significantly reduces their mobility and causes severe pain [1, 2]. Currently, there is no cure for OA and most treatment regimens focus on symptom management and pain reduction through prescription drugs [3, 4] and the use of supplements including nutraceuticals—omega 3 fatty acid glucosamine and pentosan polysulphate. Since OA is a progressive condition, it is only controlled by these drugs and supplements. Surgical options such as joint replacement are available for the hips and elbows; however, surgery is often costly. Therefore, there is an urgent need for an alternative therapy with effective disease-modifying effects.

In this regard, adult stem cell therapy, in the form of mesenchymal stem (or stromal) cells (MSCs), has recently provided a new paradigm for treating chronic arthritic dogs from symptom management to stimulating regeneration of bones and cartilage, resulting in considerable improvements in quality of life [5–14]. MSCs are acknowledged as an important new source of useful bioactive compounds with several therapeutic properties [15]. A major study by Caplan [16] demonstrated the extraordinary properties of MSCs; by complex multistep processes, these cells induced the formation of chondrocytes and osteoblast in a damaged bone. MSCs are also able to secrete a variety of bioactive factors that allow them to be attracted to the site of injury and reduce pain and inflammation and also contribute directly to the tissue repair. MSCs were able to aggregate, multiply, and bridge injured tissue by forming vasculature-controlled chondrocytes for cartilage formation and osteoblast for bone formation [16].

MSCs are derived from the mesodermal lineage and are present in the bone marrow, dermis, fat and muscle, and several other body organs as an endogenous reserve of self-renewing cells. Of these tissues, the adipose tissue provides a relatively abundant supply of MSC and is easy to isolate in greater number than the bone marrow or any other tissue type [17]. In early stages of life, each of these organs has a population of stem cells, called adult stem cells that have roles in replenishing damaged cells by self-renewal and a unique ability to form the particular cell type it resides in. With age, the supply of these self-renewing stem cells significantly decreases by tenfold from newborn to teenage years and again by teenage to old age [16].

Other important features of MSCs are their anti-inflammatory and immunosuppressive abilities enabling them to significantly attenuate the immune responses in the host [18]. These cells along with soluble bioactive compounds are then able to inhibit the activation of T lymphocytes, B lymphocytes, and natural killer cells [18–20]. Because of this important property, MSCs can be used in allogeneic treatment where the cells from one donor dog can be used to treat other dogs. With these multipotent, immune modulator properties and the ability to produce several bioactive compounds, these cells are able to regenerate and repair the cartilage and bone and provide long-term relief in the small companion animals including dogs and cats. Equine stem cells are also proven to be beneficial in treating tendon and ligament injuries in horses [17].

In this study, we report the outcome of the treatment of two hundred and three dogs with allogeneic adult stem cell therapy in dogs suffering from osteoarthritis and other joint defects. The veterinarians involved in the treatment of animals initially diagnosed and characterised the extent of degeneration by following the Kellgren-Lawrence grade system to categorise OA based on the radiographic features [25]. Furthermore, the lameness and pain scoring was carried out by the veterinarians and recorded pre- and posttreatment; scoring 1 to 5 was based on the severity of the symptoms: score of 1 for mild symptoms and score of 4 for the severe pain and lameness. Posttreatment assessment data 10 weeks after MSC treatment revealed significant improvement of OA symptoms: pain reduction, improvement of mobility, and increased daily activity as measured as quality of life score.

## 2. Materials and Methods

### 2.1. Donor Selection

The donor dogs were selected based on the following criteria: (a) less than 5 years of age, (b) satisfactory overall health and well-being based on normal clinical chemistry and haematology reports, and (c) minimal level of vaccination against DHP. Twenty grams of the adipose tissue from the abdominal region was collected from the suitable donor dogs by surgery under general anaesthesia using aseptic techniques. The adipose tissue was transported to the AVSC laboratory in a sterile transport buffer at 4 degree Celsius within four hours of collection.

### 2.2. Isolation and Purification of Adipose-Derived Mesenchymal Stem Cells

Mesenchymal stem cells were isolated, purified, and identified following the methods described in “Pittsburgh patent” AU 784580. Briefly, the adipose tissues were washed with sterile PBS buffer and subjected to enzymatic digestion to obtain stromal vascular fraction (SVF) using aseptic techniques throughout processing in a physical containment 2 (PC2) at the AVSC laboratory. The SVF cells were then expanded in a suitable growth media and cells were passaged up to passage 2. A pure culture was obtained after two passages. The cells were tested for quality and purity using clusters of differentiation (CD) markers (CD 34, 44, 45, and 90) by FACS analysis.

Australian Veterinary Stem Cell (AVSC) has been providing allogeneic culture-expanded MSCs to the veterinary profession in Australia since 2010. AVSC is a licensee of patents held by the University of Pittsburgh and Vet Stem to obtain MSCs from adipose tissue, expand MSCs in culture, and use them to treat clinical diseases. AVSC has obtained approval and complies with the Australian government regulatory authority, Australian Pesticides and Veterinary Medicine Authority (APVMA), to isolate, expand CAD-MSCs in its laboratory, and supply the cells to the requesting veterinarians treating arthritic dogs.

## 3. MSC Treatment

### 3.1. Patient Selection

A clinical examination was carried out by the treating veterinarians including a full blood examination. The joint of the dog was assessed using Table 1. The grading of OA, the age, gender, and breed were recorded. Dogs diagnosed with cancer were deemed unsuitable for the stem cell therapy. Also, any previous medication prescribed to the dog was noted. To summarise, no dogs that were treated were grade 1 on the scale; ~14% of dogs were grade 2, 53% were grade 3, and 33% were grade 4.

A prescription was sent to the AVSC laboratory with the dog's detail and dosage required. The cells were delivered to the veterinarians as per the prescription. Cell viability and number were ensured postthaw and prior to despatching the cells in a sterile syringe ready to inject.

The IA injections were administered by the veterinarians to the dogs under anaesthesia. IV injections were carried out under sedation or if patient was calm, no sedation was needed. Dogs were observed for 2 hours after receiving the injections before being allowed to return home. Owner's consent was obtained for each treated dog for a follow-up after two months posttreatment.

The dogs receiving IA or IV were placed in two separate categories. Of the 203 dogs, 128 received a single dose of IA injection in the affected joint. Sixty-five dogs received a single IV injection, and 10 dogs received both IA and IV injections. The dogs were also grouped by weight: group 1: 2–9.9 Kgs, 2: 10–20 Kgs, 3: 21–30 Kgs, and 4: 31+ Kgs.

### 3.2. Treated Dog Population

Various breeds of the dogs were treated with the allogeneic adult stem cells (CAD-MSC) including Staffordshire Terriers, Golden Retrievers, Border Collies, Siberian Huskies, Bullmastiffs, Italian Spinones, Blue Heelers, Labradors, German Shepherds, Rottweilers, Cattle Dogs, Maltese Terriers, Jack Russels, and Spoodles. The dogs were presented with OA in various joints.

The dogs were of various age groups, from 8 months to 16 years old. These were grouped in 4 different age groups A–D: group A, <5 years; group B, 6–9 years; group C, 10–12 years; and group D, 13+ years.

This study was conducted under the Monash University Animal Ethics Committee approval number SOBSA/MIS/2008/49.

### 3.3. Statistical Analysis

The frequency of each QOL score was determined; the statistical relationship between age of the dogs and QOL and weight of the dogs and QOL score were examined by a one-way between-group analysis of variance (ANOVA). Post hoc comparisons were made by the Tukey test. The statistical analysis was carried out by statistical software SPSS v 22 [21]. The QOL scores were compared among dogs of various age groups and weight groups.

## 4. Results

**Figure 1:**
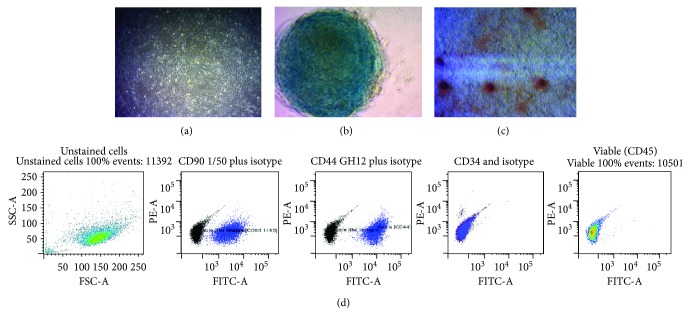
(a) Canine purified adipose MSCs at 20x magnification. (b) Differentiation assay chondrogenesis stained by Alcian blue. (c) Osteogenesis, alkaline phosphatase-positive colonies stained with Alizarin Red. (d) FACS analysis of the cultured canine adipose MSCs. Two positive and two negative CD markers for MSCs were used to identify the cells. MSCs were positive for CD90 and CD44 and negative for CD34 and CD45 cell markers.

Adipose-derived MSCs were isolated from young healthy dogs with satisfactory overall health. MSCs were cultured and expanded *in vitro* before characterisation (Figure 1(a)). The expanded cells were analysed by Fluorescence-activated cell sorting (FACS) using two positive surface markers CD44 and CD90 and two negative markers CD 34 and CD45, as shown in Figure 1(d). Furthermore, the purified and expanded cells were subjected to differentiation assays to chondrogenic and osteogenic lineages *in vitro* (Figures 1(b) and 1(c)). The results of these assays determined that the quality of the cells had met the requirements as described by the International Society of Cellular Therapy (ISCT, 2006) [22], and the cells were subsequently stored for future clinical use.

Two hundred and three dogs suffering from osteoarthritis and other joint defects were involved in the allogeneic adult stem cell therapy study. Veterinarians involved in the stem cell therapy were advised to follow the Kellgren-Lawrence grade system to categorise OA. The stage of OA was normally defined as the grade of OA based on the radiographic features as described by Table 1. The dogs treated with the CAD-MSC showed OA ranging from grade 2 to grade 4. The lameness and pain scoring was carried out by the veterinarians and recorded pretreatment; scoring 1 to 5 was based on the severity of the symptoms (Table 1). A score of 1 is given for normal lameness and no pain or functional disability through to a score of 5 for the severe pain and lameness as shown in the table.

The 203 dogs were graded for the level of osteoarthritis and the results are summarised in Table 2. A total of 32 dogs were grade 2 minimal OA, 103 grade 3 moderate OA, and 58 grade 4 severe OA (Table 2).

The outcome of the treatment of various grades of OA, in terms of improvement in pain, mobility as quality of life improvement, posttreatment was also assessed. Owner's consent was obtained by the veterinarians for a follow-up, and owners were advised to report any adverse reactions to the veterinarian immediately.

Posttreatment assessment and statistical analyses were also performed to determine the improvement in the dogs. A numeric rating scale was employed by the veterinarians based on the clinical outcome upon comparison from pre- and posttreatment questionnaires on lameness and pain reduction and improvement in mobility and overall demeanour (Table 3). The lameness and pain scoring was carried out posttreatment and compared with pretreatment scores; a quality of life (QOL) score was assigned to each dog using a score of 1 to 5 (Table 4) and restricted to only three parameters: pain, mobility, and functional disability. A score of 1 was given to the dogs exhibiting excellent improvement in terms of mobility and overall wellbeing, score 2 for good improvement, score of 3 for no difference, score of 4 for worsening of the symptoms, and score of 5 for death due to stem cells.

A total of 203 dogs were assessed for the improvement in their pain and mobility and willingness to play voluntarily. The dogs were assessed by veterinarians for the quality of life (QOL) score and the data grouped with respect to the various age groups, as well as whether they received a single dose of IA injection in the affected joint (*n* = 128), received a single IV injection (*n* = 65), and received both IA and IV injections (*n* = 10).

**Figure 2:**
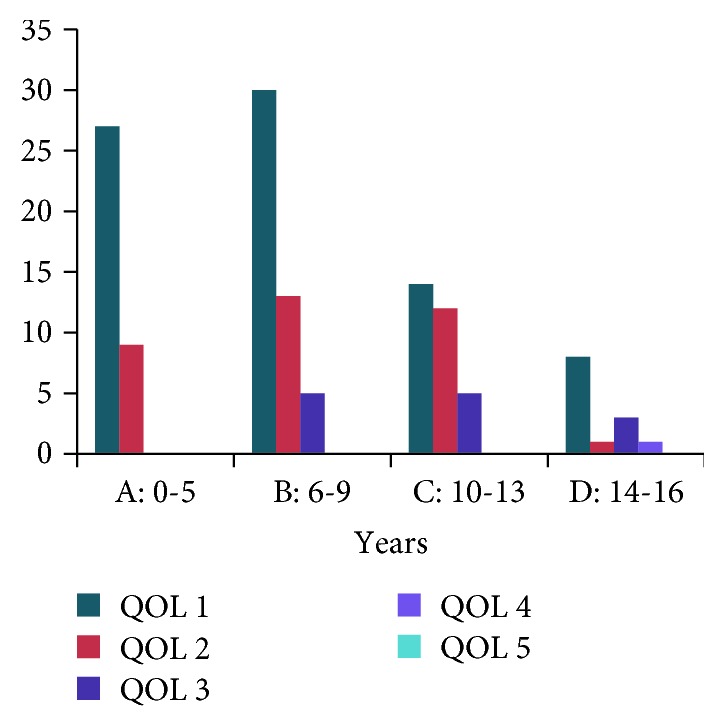
Quality of life (QOL) score for different age groups of dogs receiving single injection of MSCs by an IA injection.

Firstly, of the one hundred and twenty-eight dogs that received IA injection (Figure 2) in the affected joint, 79 were reported to have an excellent improvement (Table 5).

The number of dogs demonstrate an excellent improvement and a QOL score of 1 was statistically significant (*p* < 0.007). The QOL score between the groups was also statistically significant (*p* < 0.021). Seventy-five percent of dogs under the age of 5 (*n* = 36) had reported a QOL score of 1 suggesting excellent improvement, and 25% exhibited a QOL score of 2 suggesting good improvement. Similarly, dogs belonging to the age group 6–9 demonstrated a slightly different ratio of improvement. 62% of dogs of this age group demonstrated excellent improvement, and 27% showed good improvement, and only 6% of dogs showed no change in their condition. Overall, 89% of dogs of age 6–9 showed good improvement in their quality of life.

Dogs of age group C years showed 45% (*n* = 31) of excellent improvement, 38% of good improvement, and 16% no change in their condition after the treatment. Overall, 83% of dogs belonging to this age group showed good improvement. Similarly, in age group D, 62% of dogs (*n* = 13) reported excellent improvement, 1 dog showed good improvement, and 23% reported no change. Overall, 66.8% of dogs of this older age group showed improvements. Tables 5 and 6 demonstrate that majority of the dogs of each age group displayed a QOL score of 1 implying excellent improvement in their quality of life when compared to the other scores (Figure 2).

The results from the 65 dogs that received IV injections demonstrated the following: only 6 dogs of age group A were given IV MSCs and of that, 50% demonstrated excellent improvement; only 16.5% exhibited good improvement and 33% showed no change. Dogs belonging to the age group B demonstrated equally distributed score of 1, 2, and 3.

The dogs belonging to age group C had the largest number of dogs in it (*n* = 30) compared to any other age group. 46% of the dogs showed an excellent response, 36.6% showed good improvement, and 16.6% did not show any improvement. In age group D (*n* = 16), 19% scored QOL 1, 56.3% scored QOL2, and 25% scored QOL 3. No death has been reported for any dog undergoing MSC treatment.

Furthermore, 10 dogs received both IA and IV treatment as recommended by the veterinarian. The clinical grading for these animals and the QOL scores posttreatment are shown in Tables 7 and 8. Due to the small sample size, statistical analysis could not be performed. However, nine out of ten dogs were reported to have either good or excellent improvement (Table 8).

**Figure 3:**
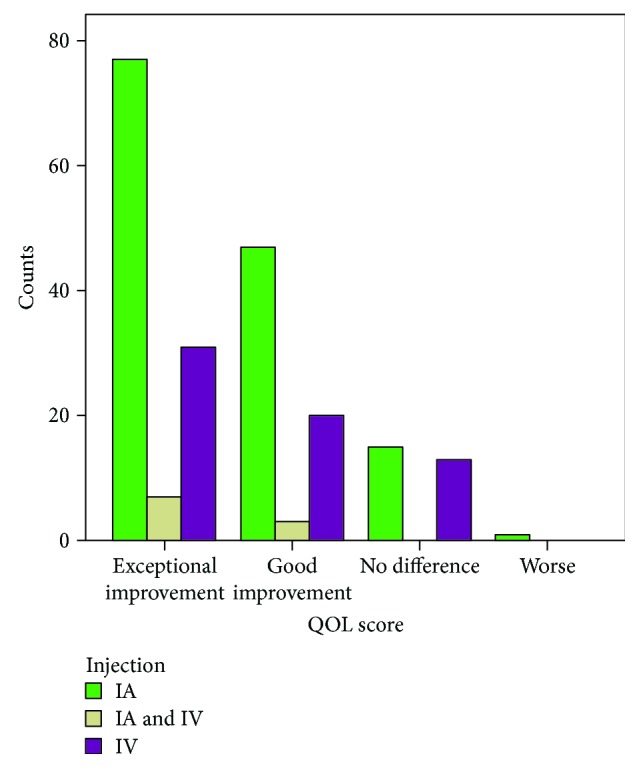
Treatment type and QOL score.

Finally, a comparison of the outcomes between all treatment types was summarised in Figure 3. Overall, it was observed that the majority of dogs showed excellent to good improvement with a QOL score of 1 or 2. The highest improvement was observed in both the IA treatment alone and the IA and IV treatment groups with ~90% of dogs receiving a QOL score of 1 or 2, while in the IV treatment-alone group, 50 out of 65 dogs (76%) showed excellent to good improvement.

## 5. Discussion

In this report, over 85% of dogs of various breeds and ages recorded combined a QOL score of 1 and 2 when assessed on the parameters of lameness and pain, suggesting significant improvement in their quality of life (*p* < 0.021). Similar results have been reported previously elsewhere [6, 7, 12]. MSC therapies have been attributed as being one of the major breakthroughs in treating osteoarthritic conditions in dogs and other animals. It is safe and effective and employs endogenous repair within the body helping osteoarthritic dogs to improve quality of life significantly [7–9, 11, 12, 16]. MSCs are known to differentiate into various tissue types, aid the body's own regeneration abilities, and also produce several useful bioactive compounds that assist in repairing damaged tissues and are capable of regeneration of degenerated tissues [15]. Due to the anti-inflammatory properties and immune modulation capabilities, MSCs are safe to use in recipients without causing any immune response and other adverse effects [18, 20]. The result from our own experiences stated in this report supports the similar findings by other workers [6–9, 11–13, 22, 23].

The improvement on QOL scores was better in dogs under the age of 9 (Figure 2), when compared to the older dogs. The QOL score of 3 (no change) could be attributed to the advanced stage of the disease, severe degenerative alterations in bone architecture, and other contributing factors such as poor health and prolonged diseased state. There was one case reported where the QOL was scored 4 for a dog 14 years of age that had multiple joints affected including both hips and stifles. The dog did not respond to the treatment and had shown worsening in condition but was managed on NSAIDs. The natural progression of the disease for this particular dog was observed.

Of all the dogs undergoing CAD-MSC therapy, none displayed any severe adverse events other than slight discomfort; two of the dogs exhibited a mild skin allergy which was managed on antiallergic medication.

As observed in Figure 3, IA treatment provided better results when compared with the IV treatment. However, IV treatment was only chosen because of the polyarthritis condition, and this could have attributed to the lower improvement. Also, it is worth noting that the age group played a major role in achieving the best outcomes. Most of the dogs receiving IA treatment under the age of 5 showed good improvement and therefore suggested that the dog's own overall health and vitality are significant factors in response to the MSC therapy [24–26].

In conclusion, the innovative allogeneic CAD-MSC treatment provides good alternative treatment modality in the management of canine osteoarthritis. The long-term assessment and ongoing reporting of the larger population of dogs undergoing stem cell therapy is warranted to further understand the therapeutic benefits of this new technology.

## Figures and Tables

**Table 1:** The Kellgren-Lawrence grading of osteoarthritis^∗^.

Grading	Radiographic features	OA
Grade 0	No abnormalities	No features of OA
Grade 1	Minute osteophytes	Doubtful
Grade 2	Definite osteophytes	Minimal
Grade 3	Diminished joint space	Moderate
Grade 4	Greatly diminished joint space + sclerosis of the subchondral bone	Severe

^∗^Adapted from Arden and Nevitt [25], *Best Practice and Research Clinical Rheumatology*.

**Table 2:** Osteoarthritis grade evaluated by the vets prior to CAD-MSC treatment.

Age group	Grade 2	Grade 3	Grade 4
A: 0–5 years	14	14	14
B: 6–9 years	15	40	6
C: 10–13 years	3	42	16
D: 14–16 years	0	7	22

**Table 3:** Symptoms of arthritic knee; lameness and pain scores^#^.

Lameness	Pain	Functional disability
1 (normal)	1 (no pain)	1 (normal)
2 (intermittent)	2 (mild pain)	2 (slightly stiff)
3 (persistent)	3 (severe pain)	3 (stiff)
4 (non-weight bearing)	4 (severe pain)	4 (very stiff, unwilling to walk)
5 (ambulatory only with assistance)	5 (severe pain)	5 (need assistance to walk)

^#^Adapted from Black et al. [6].

**Table 4:** Quality of life (QOL) score.

Score	Symptoms
1	Excellent improvement
2	Good improvement
3	No difference
4	Worse
5	Considerably worse or death

**Table 5:** IA injection and quality of life score with respect to the age groups.

Age group	QOL 1	QOL 2	QOL 3	QOL 4	QOL 5
A: 0–5 years	27	9	0	0	0
B: 6–9 years	30	13	5	0	0
C: 10–13 years	14	12	5	0	0
D: 14–16 years	8	1	3	1	0

**Table 6:** IV injection and quality of life score with respect to the age groups.

Age group	QOL 1	QOL 2	QOL 3	QOL 4	QOL 5
A: 0–5 years	3	1	2	0	0
B: 6–9 years	5	4	4	0	0
C: 10–13 years	14	11	5	0	0
D: 14–16 years	3	9	4	0	0

**Table 7:** Osteoarthritis grade evaluated by the vets prior to CAD-MSC treatment for dogs receiving both IA and IV injections.

Age group	Grade 2	Grade 3	Grade 4
A: 0–5 years	2	1	1
B: 6–9 years	0	1	1
C: 10–13 years	0	0	1
D: 14–16 years	0	2	1

**Table 8:** IA and IV injection and quality of life score with respect to the age groups.

Age group	QOL 1	QOL 2	QOL 3	QOL 4	QOL 5
0–5 years	2	2	0	0	0
6–9 years	1	0	1	0	0
10–13 years	1	0	0	0	0
14–16 years	2	1	0	0	0

## Abbreviation

CAD-MSCs: Canine adipose-derived mesenchymal stem cells
OA: Osteoarthritis
QOL: Quality of life.
